# Attitude changes following short-form opioid overdose video education: a pilot study

**DOI:** 10.1186/s12954-022-00696-4

**Published:** 2022-10-14

**Authors:** Mika V. Galiher, Miranda Huffman

**Affiliations:** 1 Meharry Medical College, 1005 Dr. D.B. Todd Jr. Blvd., Nashville, TN 37208-3599 USA; 2 Department of Family and Community Medicine, Meharry Medical College, 1005 Dr. D.B. Todd Jr. Blvd., Nashville, TN 37208-3599 USA

**Keywords:** Naloxone training, Overdose, Opioid, Prevention, Video education, Accessibility

## Abstract

**Background:**

Opioid overdose response training (OORT) and the need for its rapid expansion have become more significant as the opioid epidemic continues to be a health crisis in the USA. Limitation of funding and stigmatization often hinders expansion of OORT programs. Primarily due to the COVID-19 pandemic, there has been widespread transition from in-person to virtual communication. However, OORT programs may benefit from long-term use of this modality of education if it can be as effective.

**Objective:**

To measure the change in participant attitude after a brief, virtual OORT.

**Methods:**

A 6.5-min OORT video explained recognition of opioid overdose, appropriate response and proper administration of intranasal naloxone. Pre- and post-video scores from a 19-item survey were used to determine the video's impact on participants’ self-perceived competence and readiness to administer naloxone to a person with a suspected opioid overdose. Paired *t* tests were used in the analysis of pre- and post-video scores. Mann–Whitney U and Kruskal–Wallis H testing were used to compare variance between several demographic subgroups of interest.

**Results:**

A sample of 219 participants had a significant mean difference of 15.12 (SD 9.48; 95% CI 13.86–16.39, *p* < 0.001) between pre- and posttest scores. Improvements were found to be greatest in content-naïve participants with lower levels of education and non-health care-related jobs than participants endorsing previous content awareness, formal naloxone training, masters, doctorate or professional degrees and health care-related jobs.

**Conclusion:**

This pilot study demonstrated encouraging evidence that a brief, virtual, pre-recorded educational intervention improved participant-rated competence and readiness to administer intranasal naloxone in a suspected opioid overdose. Due to scalability and ability to overcome common healthcare accessibility barriers, short-form videos focused on key facts about naloxone and the benefits of its use could be part of a strategy for rapid expansion of OORT programs to mitigate opioid overdose fatalities.

## Background

The opioid epidemic in the USA has persisted for decades and markedly worsened within the past few years [1]. Despite numerous continuous public health efforts to mitigate misuse and overdose, opioid overdose fatalities increased from 50,963 in 2019 to 69,710 in 2020 [2]. The almost 37% increase has been attributed to mounting socioeconomic pressures and increasingly limited access to health care, secondary to the COVID-19 pandemic and compounded by the surge in synthetic opioids [3]. Evidence has suggested that opioid overdose education and distribution of naloxone to lay-persons and heroin users are cost-effective strategies at reducing opioid overdose deaths [4]. Efficacy of varied methods of opioid overdose response training (OORT) has been an area of flourishing research [5, 6]. A variety of in-person, online and hybrid training programs ranging from 10 min to 4 h have proven effective in teaching recognition of signs and symptoms of an opioid overdose and proper administration of naloxone [7, 8].

In the field of OORT research, the Opioid Overdose Knowledge Scale (OOKS) and Opioid Overdose Attitudes Scale (OOAS) have become standard pre- and post-educational intervention measures [9, 10]. The OOKS and OOAS were developed in 2013 using a sample of 42 family members or friends of people with an opioid use disorder (OUD) and 56 healthcare professionals [11]. The initial survey and a 7-day repeated measure were shown to have internal reliability, construct validity and test–retest reliability. Between both groups, healthcare professionals scored significantly higher than family and friends of people with an OUD across both scales.

In 2015, a study, using the OOAS as their pre-posttest measure, included 428 participants and studied score changes following a 90-min in-person OORT [12]. Participants were trained in either intramuscular (IM) or intranasal (IN) naloxone. The results indicated significant improvement following the OORT, and more specifically, participants trained in IN rather than IM naloxone scored significantly higher in self-perceived confidence on posttest. It is worth considering whether the lengthy training required to explain IM naloxone administration is still necessitated, since naloxone has shifted from IM to primarily IN administration (Narcan) in recent years. The transition has both increased ease of administration and decreased risk of needlestick injury [13]. Additionally, participants in this study identifying as either a healthcare provider, or a family member or friend of a person with an OUD scored significantly higher than other participants.

Virtual health education has proven successful in improving medical outcomes in areas void of conventional resources with minimal cost comparative to in-person educational programs [14]. Additionally, since the onset of the COVID-19 pandemic, the appeal of virtual health education and telemedical interventions for patients with substance use disorders has increased as a method to safely social distance [15]. Video education on other health topics (e.g., outcomes in heart failure, prostate cancer screening and CPR instruction) has been shown to have comparable results in content testing and medical outcome to in-person education [16–18]. It is worth noting that the opioid epidemic is not unique to urbanicity [19]. However, outreach programs and naloxone access continue to remain disproportionately more available in metropolitan rather than rural regions of the country [20, 21]. Fewer resources at farther distances in rural regions often create barriers to healthcare access. With these considerations, measuring the impact of a brief video education provided virtually on participants’ attitudes toward overdose could be a significant step toward future mitigation of opioid use morbidity and mortality. Decreasing financial burden of educational programs while increasing scalability has become ever more significant as opioid fatalities and the demand for naloxone continue to rise [7, 13].

It has been shown that online OORT programs can be equally as effective as in-person OORT programs in a population of medical school students [22]. In another study, either in-person or through a live, online platform, ranging from 2–3 h a sample of 381 participants demonstrated improvement in OOAS items, along with measures of knowledge and stigma toward opioid use disorder (OUD) [23]. It should be noted, however, that the majority of the study participants were graduate students in health-related fields, which limits the generalizability of the findings.

In consideration of earlier studies, it is readily apparent that significant research has been done around online versus in-person opioid overdose education and its effects on certain demographic subgroups. However, there has yet to be a study with an educational intervention less than 20 min, without a live instructor [22, 24].

There is a body of research that points to the effectiveness of in-person opioid overdose education [7, 8, 12, 13]. However, there is a recognition that virtual education and telemedical interventions have the potential to also be an effective means of delivering OORT [15]. Previous research that has examined the effectiveness of virtual OORT [14, 15] has relied on presentations of at least 1 h; as such, the current study aimed to pilot test a brief (< 10 min), online video intervention. Specifically, the primary aim of this pilot was to determine the efficacy of this brief, online video on opioid overdose and naloxone administration would significantly improve participants’ attitudes to respond to and administer intranasal naloxone in an opioid overdose situation.

## Method

### Trial design and recruitment

A non-randomized, single (intervention only) arm pre-posttest study was used to assess the efficacy of a brief online video education on participants’ attitudes toward opioid overdose. Participants were recruited through convenience and snowball sampling through posts and reposts on social media, with the highest visibility repost by an addiction interest forum on Facebook, “The Addict’s Diary”. Members of this forum were interested in addiction in some way (e.g., academic interest, personally in recovery from a SUD, had a family with a SUD, etc.). Additionally, the senior author (MH) contacted colleagues working in family medicine who advertised the study in their waiting rooms. No exclusion criteria were used during participant recruitment (e.g., healthcare workers who may have been familiar with the material presented in the video were not excluded). A target sample size of 200 was set based on previous OORT pre-posttest research and suggested guidelines for quasi-experimental studies [25–29]. The study protocol was approved by the Internal Review Board at Meharry Medical College.

### Informed consent and participant data collection

Access to the survey was provided through a link and administered using Typeform. The landing page of the link was the informed consent document with a risk warning indicating that the following content may be upsetting for some people and would include discussion of opioid overdose and fatalities. To advance in the study, participants were required to click “I understand and agree.” All data were collected anonymously and Typeform automatically removed all incomplete data sets. Participants did not receive any compensation and were able to discontinue the survey at any point by exiting their browser window. Pretest and posttest items including items related to demographics were forced response. All demographic items that were forced response included the options “prefer not to say” and “other” with a free-form text input box, with the gender options also including a “male,” “female,” and “non-binary” option. Ethnicity options were taken from the 2020 US Census [30].

### Instrument

A 19-item survey was adapted from the 28-item Opioid Overdose Attitudes Scale (OOAS), which has internal (Cronbach’s alpha = 0.90) and test–retest reliability (intraclass correlation = 0.82) [11]. Permission for use was attained from Dr. Anna Williams of Kings College London. Participants received the same 19-item survey as both pre- and posttest. Questions related to demographics and base familiarity with subject matter preceded only the pretest. Items are rated on a Likert scale from 1 = strongly disagree to 5 = strongly agree. The original 28-item OOAS is divided into three subscales: competence, concerns, and readiness.

Since online surveys suffer from higher attrition rates with an inverse relation to time required for completion, the survey was abbreviated [31]. Retaining only items from the competence and readiness subscales allowed maintenance of the study objective while decreasing completion time to approximately 15 min. Since the concerns subscale was removed from the abbreviated survey, the two remaining dimensions, competence and readiness, comprise the attitude toward opioid overdose construct in this study. All survey items are listed in Table 4. Items discussing intramuscular naloxone were modified for the use of intranasal naloxone and 2 items that covered topics beyond the scope of the video were removed from the competence subscale.

### Intervention

A 6.5-min educational video was created in a tutorial-style format using animated graphic overlays (https://youtu.be/9hw6E9389W8). Following completion of the pretest, participants were prompted to click on a link to the 6.5-min educational video. The video was intended to be easily understood by an audience at any level of formal education, regardless of previous knowledge of the subject. Material included in this video was based on a summary of multiple OORT videos and presentations [32–36]. Informational content covered recognition of signs and symptoms of an opioid overdose, appropriate response and proper administration of intranasal naloxone. The video concept outline, transcript, and graphics are included in Supplementary Materials. The video also incorporated a stigma reduction component by concluding with a brief testimonial of an opioid overdose survivor who had been resuscitated with naloxone [37].

### Analysis

A paired *t* test was used to evaluate the statistical significance of changes between pre- and posttest scores of: (1) the scale of attitude toward opioid overdose, (2) the subscale of competence, (3) the subscale of readiness, and (4) individual survey items. Mann–Whitney U tests and Kruskal–Wallis H tests were conducted on pre- and posttest results separated by various demographic subgroupings to determine homogeneity of variance between groups. Demographic subgroups with suspected increased health literacy, increased base knowledge or increased interest in the subject material were specifically isolated for analysis of variance between groups. A standard statistical significance of *p* < 0.05 was used throughout analysis.

Analysis was completed using IBM SPSS statistics program. All text answers were operationalized, and four negative items’ results were reverse coded in SPSS [38]. Characteristics of the sample were assessed using descriptive statistics of demographic question results.

## Results

### Participant characteristics

A total of 219 participants completed the survey in its entirety. An additional 129 participants discontinued the survey immediately after agreeing to the informed consent and only 25 participants partially completed the survey. Table 1 presents the demographic characteristics of the 219 participants that completed the survey.

**Table 1:** Participant characteristics

Characteristic	Participants, *n* (%)
*Age (years)*	
18–24	25 (11.4)
25–34	69 (31.5)
35–50	56 (25.6)
51–69	57 (26.0)
70 +	12 (5.5)
*Sex*	
Male	63 (28.8)
Female	156 (71.2)
Potential familiarity with subject matter	
*Do you work in health care?*	
Yes	65 (29.7)
No	154 (70.3)
*Have you had any personal experience with addiction/substance abuse? (e.g., yourself, family member or a close friend)*	
Yes	154 (70.3)
No	65 (29.7)
*Have you had any personal experience with opioid/opiate abuse? (e.g., yourself, family member or a close friend)*	
Yes	101 (46.3)
No	118 (53.7)
*Do you know what naloxone (Narcan) is?*	
Yes	163 (74.4)
No	56 (25.6)
*Have you ever had any naloxone (Narcan) training?*	
Yes	43 (19.6)
No	176 (80.4)
*Ethnicity*	
White	155 (70.8)
Black/African-American	31 (14.2)
Asian	18 (8.2)
Hispanic, Latino/Spanish-origin	12 (5.5)
Middle Eastern/North-African	9 (4.1)
Native-American or Alaska Native	2 (0.9)
Prefer not to say	8 (3.87)
*Level of education*	
High school	29 (13.2)
Vocational training	12 (5.5)
College	105 (47.9)
Masters	36 (16.4)
Doctorate	18 (8.2)
Professional degree	19 (8.7)

### Effects of video education on opioid overdose attitudes

Participants demonstrated a significant improvement in attitude toward opioid overdose management following the 6.5-min educational video (mean difference = 15.12 ± 9.49; *t* (218) = 23.59, *p* < 0.001, *d* = 1.59). Separated by subscale, self-perceived competence (mean difference = 12.98 ± 8.32; *t* (218) = 23.07, *p* < 0.001, *d* = 1.56) showed greater improvement than self-perceived readiness post-video (mean difference = 2.15 ± 2.99; *t* (218) = 10.63, *p* < 0.001, *d* = 0.78). Full results for each item are presented In Table 2.

**Table 2:** Survey items with mean pre- and posttest score

Survey item	Pretest mean (SD)	Posttest mean (SD)	*p* value
*Subscale: competence*			
I have enough information about how to manage an overdose	2.23 (1.28)	4.43 (0.71)	< 0.001
I would be able to administer naloxone to someone who has overdosed	2.39 (1.56)	4.63 (0.65)	< 0.001
I would be able to check that someone who had an overdose was breathing properly	3.20 (1.44)	4.48 (0.76)	< 0.001
I am going to need more training before I would feel confident to help someone who had overdosed.*	2.11 (1.38)	3.62 (1.26)	< 0.001
I would be able to perform CPR on someone who had overdosed	3.54 (1.40)	4.17 (1.05)	< 0.001
I would be able to perform chest compressions on someone who had overdosed	3.74 (1.34)	4.42 (0.89)	< 0.001
If someone overdoses, I would know what to do to help them	2.86 (1.35)	4.47 (0.76)	< 0.001
I know very little about how to help someone who has overdosed.*	2.90 (1.45)	4.31 (0.92)	< 0.001
I would be able to deal effectively with an overdose	2.82 (1.29)	4.23 (0.78)	< 0.001
*Subscale: readiness*			
Family and friends of drug users should be prepared to deal with an overdose	4.62 (0.68)	4.75 (0.57)	< 0.001
If I witnessed an overdose, I would call an ambulance straight away	4.87 (0.41)	4.90 (0.39)	0.179
If I saw an overdose, I would panic and not be able to help.*	4.26 (0.88) **1.74 (0.88)	4.52 (0.83) **1.48 (0.83)	< 0.001
I would stay with the overdose victim until help arrives	4.73 (0.60)	4.85 (0.42)	< 0.001
If I saw an overdose, I would feel nervous, but I would still take the necessary actions	4.24 (0.90)	4.49 (0.79)	< 0.001
I will do whatever is necessary to save someone’s life in an overdose situation	4.51 (0.80)	4.66 (0.68)	< 0.001
If someone overdoses, I want to be able to help them	4.73 (0.60)	4.81 (0.50)	0.008
Everyone at risk of witnessing an overdose should be given a naloxone supply	3.95 (1.13)	4.60 (0.77)	< 0.001
I couldn’t just watch someone overdose, I would have to do something to help	4.70 (0.60)	4.77 (0.56)	0.075
If someone overdoses, I would call an ambulance but I wouldn’t be willing to do anything else.*	3.94 (1.25)	4.33 (1.13)	< 0.001
19-item score	70.33 (11.35)	85.46 (8.77)	< 0.001

*Reverse-coded items, **Score before reverse-coding for comparison

**Fig. 1:**
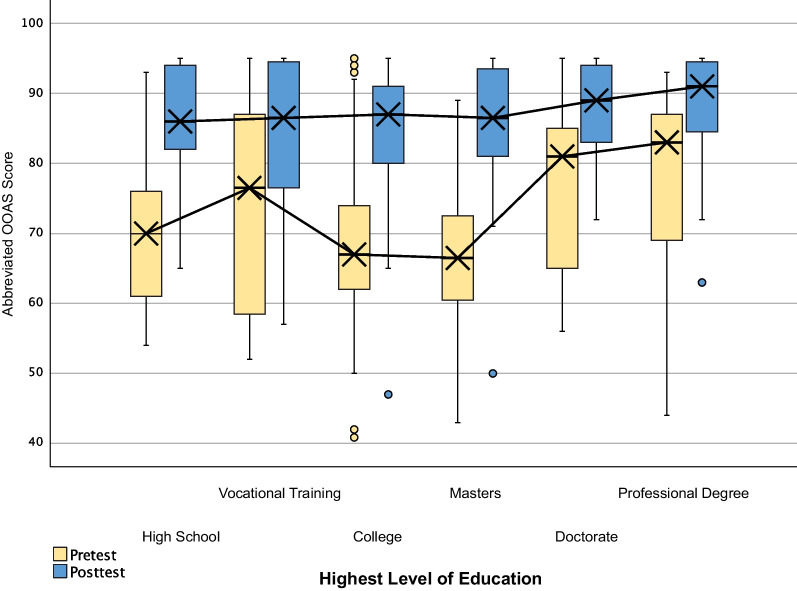
Box and whisker plots of pre- and posttest score comparison, separated by participant-identified highest level of education attained. Lines show differences between subgroups from pretest to posttest

Significant differences between educational subgroups’ medians were noted in pretest scoring, but were found to be nonsignificant in posttest scoring (Table 3; Fig. 1). Participants with doctorate and professional degrees demonstrated less improvement than lower levels of education, with the exception of vocational training. A box and whisker plot demonstrates pre- and posttest scores divided by highest level of education attained (Fig. 1).

**Table 3:** Kruskal–Wallis H Test comparison of differences between medians of groups separated by highest level of education, pretest and posttest

Subgroup variable	Groups	N	Mean rank		Kruskal–Wallis H	*p*-value
Level of education	High school	29	112.98	Pretest	17.21	0.004
	Vocational training	12	119.42			
	College	105	98.89			
	Masters	36	96.54			
	Doctorate	18	140.06			
	Professional	19	152.42			
	High school	29	108.50	Posttest	3.31	0.653
	Vocational training	12	109.29			
	College	105	104.41			
	Masters	36	111.88			
	Doctorate	18	120.36			
	Professional	19	130.26

**Fig. 2:**
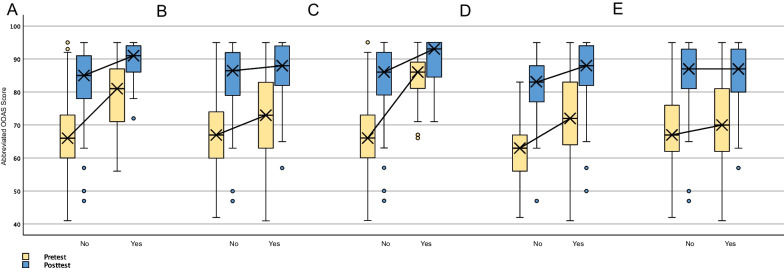
Box and whisker plots of pre- and posttest score comparisons, separated by participant-identified demographic subgroups. Lines show differences between subgroups from pretest to posttest. **A**. Does not work in health care vs. works in health care, **B**. No personal experience or relationships with opioid abuse vs. personal experience or relationships with opioid abuse, **C**. Not familiar with naloxone vs. familiar with naloxone, **D**. No previous naloxone training vs. previous naloxone training, **E**. No personal experience or relationships with substance abuse vs. personal experience or relationships with substance abuse

The same pattern was found between participants who endorsed a personal relationship or personal experience with opioid abuse (Table 4; Fig. 2b). Significant differences between pretest scores medians in healthcare and non-healthcare workers, in those with and without previous naloxone training, and in those with and without previous familiarity with naloxone were maintained in posttest scores (Table 4; Fig. 2a, c, d). However, all showed a decrease in magnitude of difference, as indicated by z-scores from pre- to posttest score. No significant differences were found between pre- or posttest scores in subgroups divided by personal relationship and/or personal experience with substance abuse (Table 4; Fig. 2e).

**Table 4:** Comparison of differences between medians of demographic subgroups pre- and posttest

Subgroup variable	Groups	*N*		Mean rank	U score	*z*-score	*p*-value
Personal experience or relation to opioid abuse	No	118	Pretest	96.33	4346.50	− 3.45	0.001
	Yes	101		125.97			
	No	118	Posttest	103.42	5183.00	− 1.66	0.096
	Yes	101		117.68			
Employment in health care	No	154	Pretest	91.11	2095.50	− 6.80	0.000
	Yes	65		154.76			
	No	154	Posttest	96.83	2977.50	− 4.74	0.000
	Yes	65		141.19			
Previous naloxone training	No	176	Pretest	92.49	701.50	− 8.28	0.000
	Yes	43		181.69			
	No	176	Posttest	101.57	2301.00	− 3.99	0.000
	Yes	43		144.49			
Familiarity with naloxone	No	56	Pretest	63.50	1960.00	− 6.37	0.000
	Yes	163		125.98			
	No	56	Posttest	83.28	3067.50	− 3.66	0.000
	Yes	163		119.18			
Personal experience or relation to substance abuse	No	65	Pretest	101.72	4467.00	− 1.25	0.209
	Yes	154		113.49			
	No	65	Posttest	108.91	4934.00	− 0.17	0.868
	Yes	154		110.46

## Discussion

Naloxone awareness and education have not been able to match the tragic level of ubiquity attained by opioid use and related fatalities in America. As the extraordinary number of these preventable deaths continues to grow, innovative solutions need to be considered to hasten expansion of OORT programs. The results of this pilot study provide encouraging initial support for the efficacy of short-form video education in improving participant self-perception of ability to manage opioid overdoses. Although social distancing due to COVID-19 was the initial motivation for the study on virtual opioid overdose education, the results have implications beyond the pandemic. Statistically significant improvements across both subscales following the 6.5-min video are congruent with the improvements following the previously discussed in-person educational interventions.

Despite the vast discrepancy in duration of educational intervention, improvements in overlapping OOAS items between current study and the 2022 Bascou et al. study using an in-person or live, online OORT of 2–3 h in duration and this study were comparable [23]. However, it is worth noting the OUD study trained and tested participants on opioid overdose knowledge and stigma toward OUD in addition to attitude toward opioid overdose and naloxone administration. While markedly shorter, this study’s 6.5-min video covered fewer topics and all survey items were focused on assessing attitudes toward intervention. Despite the differences between studies, the informal comparison is still favorable and encourages further exploration.

### Subgroups

The results of certain subgroups were selected for comparison due to suspected correlation to health literacy and previous exposure to video content [12]. Decrease in variance between subgroups from pre- to posttest suggests that the video intervention may function as an equalizer in populations that initially present with a large score differential in pretest. Participants denying previous naloxone training, personal experience or relationship with opioid abuse, and employment in health care showed significantly greater improvement following the short-form online video education than those endorsing previous naloxone education, personal experience with opioid abuse and healthcare employment, respectively. This is likely due to the fact that the participants with prior knowledge/experience with naxolone and opioids had higher scores at pretest, indicating that they already showed a reasonably high degree of comfort in recognizing and intervening during an opioid overdose.

### Limitations

Although the aim of this pilot study was to test the efficacy of the brief video, there were several limitations that temper the conclusions that can be drawn. First, an in-person control group would allow direct comparisons to fully determine the efficacy of this video. Second, the current study did not use any eligibility criteria to exclude anyone with prior training in overdose prevention, which may have confounded the results. Last, previous research also measured participant engagement and satisfaction to determine whether they actually watched the video and if they found it useful. At this point in time, the results of this pilot study are not conclusive due to these limitations; however, the results are encouraging, given the large effect sizes observed. Future research using a randomized control group, eligibility criteria, and measurements of engagement and satisfaction are necessary to determine if this intervention translates into a substantial and effective change in attitude.

## Conclusion

The training was not only brief, but also prerecorded and still yielded encouraging initial results. Moving from in-person and live to virtual and prerecorded education could scale distribution of OORT programs, reducing fiscal burden. Transitioning OORT programs toward shorter, virtual education could also allow for more expansive program coverage. Virtual OORT programs could potentially decrease accessibility issues. Additionally, patient avoidance due to perceived stigma, scheduling, and transportation show improvement with online transitioning [39]. It should be noted, however, that in-person can also be advantageous over virtual programs in some contexts: for example, it is likely that some important hands-on interaction and co-learning experiences will be lost in virtual classes. As such, both in-person and virtual media may have their place in interventions.

This pilot study provides initial evidence that pre-recorded, virtual educational interventions of extremely brief duration could be as effective as longer in-person seminars in improving attitudes toward opioid overdose. The need to decrease opioid overdose fatalities in cost-effective ways cannot be overstated. The COVID-19 pandemic has simultaneously increased use of illicit substances and isolation, which has led to increasing opioid overdose fatalities [1]. Implementation and expansion of OORT programs are proven strategies to prevent deaths from opioid overdoses [40]. Despite multiple limitations of this pilot study, the results suggest that short-form educational videos could be a fundamental part of a future strategy to rapidly scale OORT programs, increasing their potential reach to decrease opioid overdose fatalities.

## Data Availability

The dataset supporting the conclusions of this article and supplementary materials (including the video concept, transcript and.mov of the video) are available in the Mendeley Data repository, Galiher, Mika (2022), “Attitude Changes Following Brief Opioid Overdose Video Education: A Pilot Study”, Mendeley Data, V3, https://doi.org/10.17632/ngv538r68c.3. Video: A Youtube link to the educational intervention video is included within the manuscript text (https://youtu.be/9hw6E9389W8).

## Abbreviations

OORT: Opioid overdose response training
SD: Standard deviation
CI: Confidence interval
OUD: Opioid use disorder
OOKS: Opioid overdose knowledge scale
OOAS: Opioid overdose attitudes scale
IM: Intramuscular
IN: Intranasal
